# Supplementary figures and images for: Structure of the human C9orf72-SMCR8 complex reveals a multivalent protein interaction architecture
Source: PLoS Biol. 2021 Jul 23;19(7):e3001344. doi: 10.1371/journal.pbio.3001344 (PMC8336837; doi:10.1371/journal.pbio.3001344)

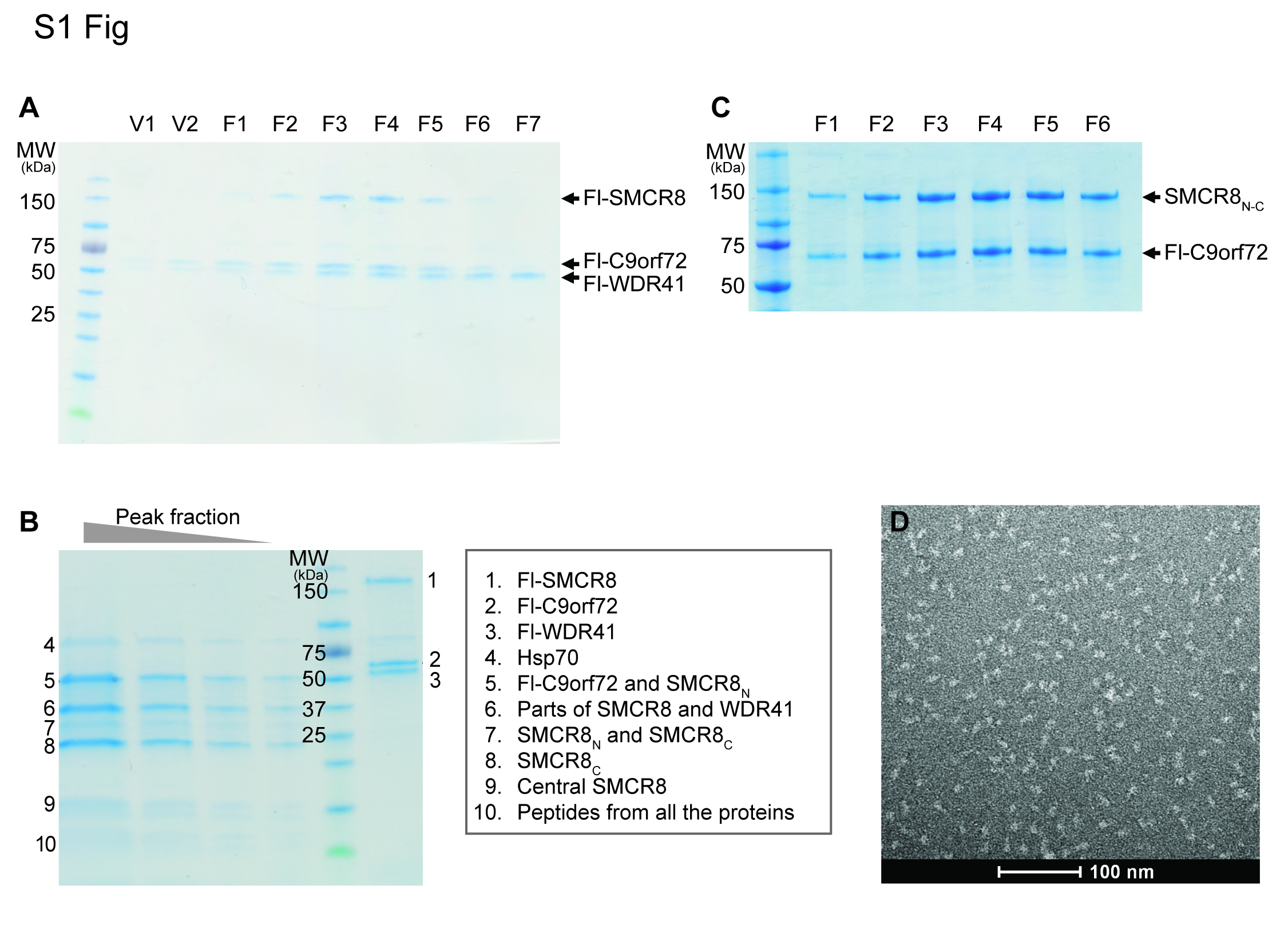

Supplement: S1 Fig — (A) Original image of the Coomassie stained SDS-PAGE gel (peak fraction F3 shown in Fig 1A) analyzing the fractions from the size-exclusion chromatography of the trimeric full-length C9orf72 complex. (B) Coomassie stained SDS-PAGE gel analyzing the peak fraction of the proteolyzed trimeric C9orf72 complex obtained after size-exclusion chromatography. The bands (numbered from 1 to 10) were subsequently excised and analyzed by mass spectrometry. The identity of each band is described on the right. (C) Original image of the Coomassie stained SDS-PAGE gel (peak fraction F4 shown in Fig 1B) analyzing the fractions from the size-exclusion chromatography of the C9orf72-SMCR8N-C complex. (D) Negative-stain micrograph of the C9orf72-SMCR8N-C complex showing homogenous distribution. Fl, full-length. (TIF) [file pbio.3001344.s001.tif]

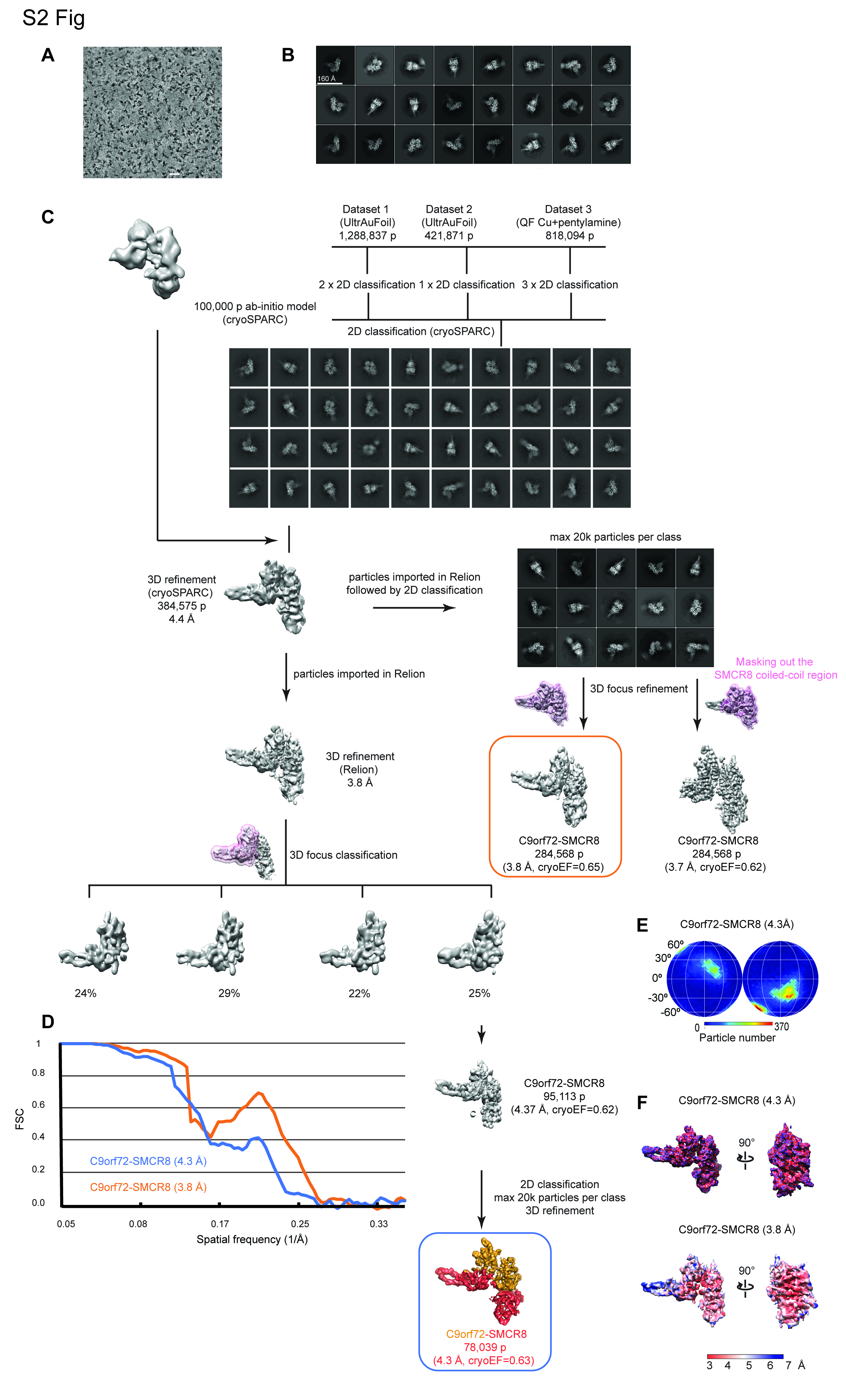

Supplement: S2 Fig — (A) Representative cryo-EM micrograph after denoising with JANNI [66]. (B) Reference-free 2D class averages for the C9orf72-SMCR8N-C complex. (C) Data processing scheme (see Materials and methods: “Cryo-electron microscopy image processing”). (D) Gold-standard FSC curves for the 3D reconstructions used for model building and interpretations. The raw data can be found in S1 Data. (E) Angular distribution of the particles used for reconstruction for the map obtained at 4.3 Å resolution. (F) Local resolution filtered maps (MonoRes, XMIPP [67]) colored according to the local resolution. cryo-EM, cryo-electron microscopy; FSC, Fourier shell correlation. (TIF) [file pbio.3001344.s002.tif]

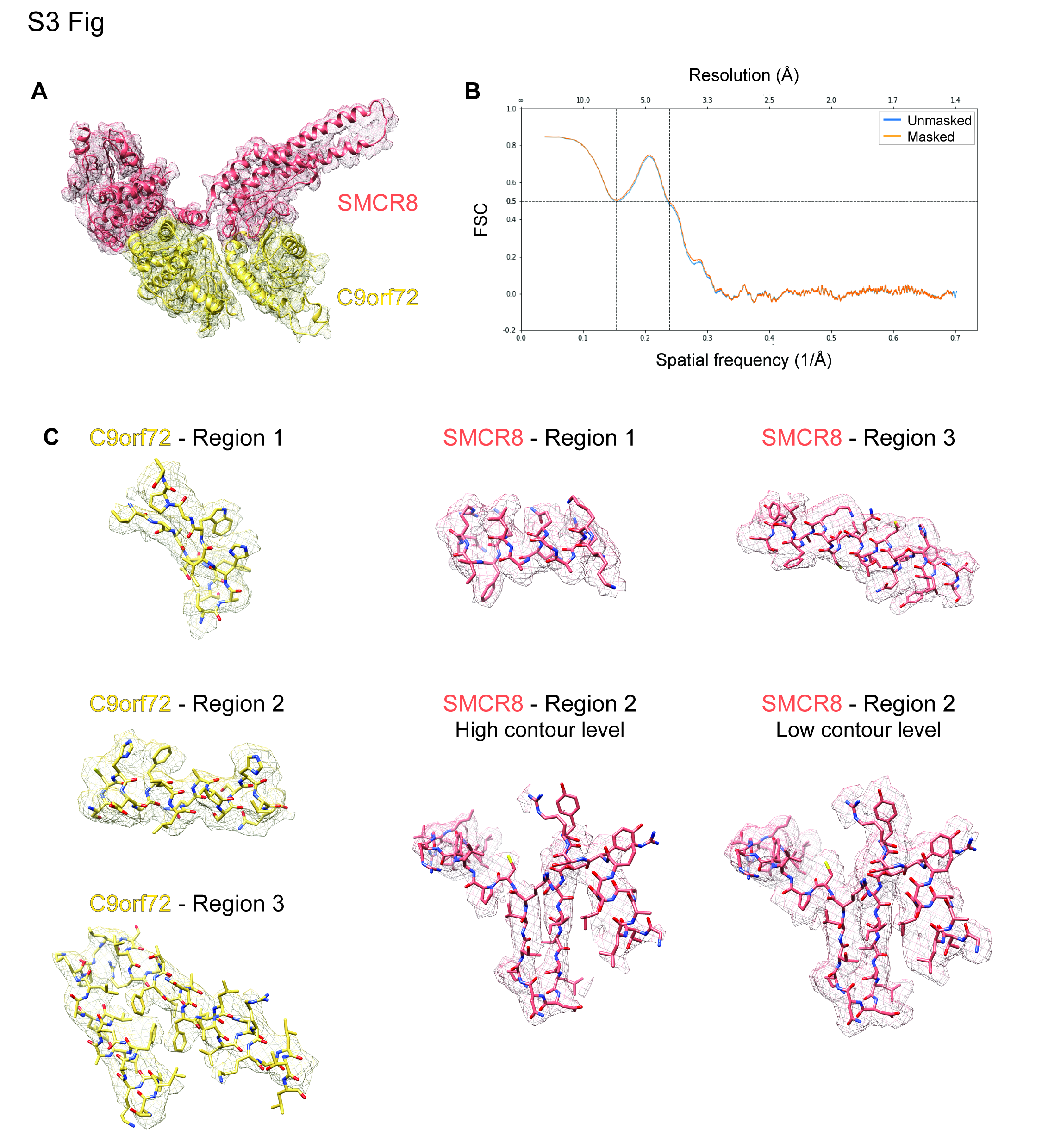

Supplement: S3 Fig — (A) Overall density fit for C9orf72 (yellow orange) and SMCR8 proteins (salmon pink). (B) Map to model FSC curve. The raw data can be found in S1 Data. (C) Images showing well-resolved regions in the cryo-EM map of C9orf72 (yellow orange) and SMCR8 (salmon pink). Residues are the following: C9orf72 Region 1: 26–31 and 41–48; C9orf72 Region 2: 214–229; C9orf72 Region 3: 375–412; SMCR8 Region 1: 703–716; SMCR8 Region 2: 795–830; SMCR8 Region 3: 841–862. cryo-EM, cryo-electron microscopy; FSC, Fourier shell correlation. (TIF) [file pbio.3001344.s003.tif]

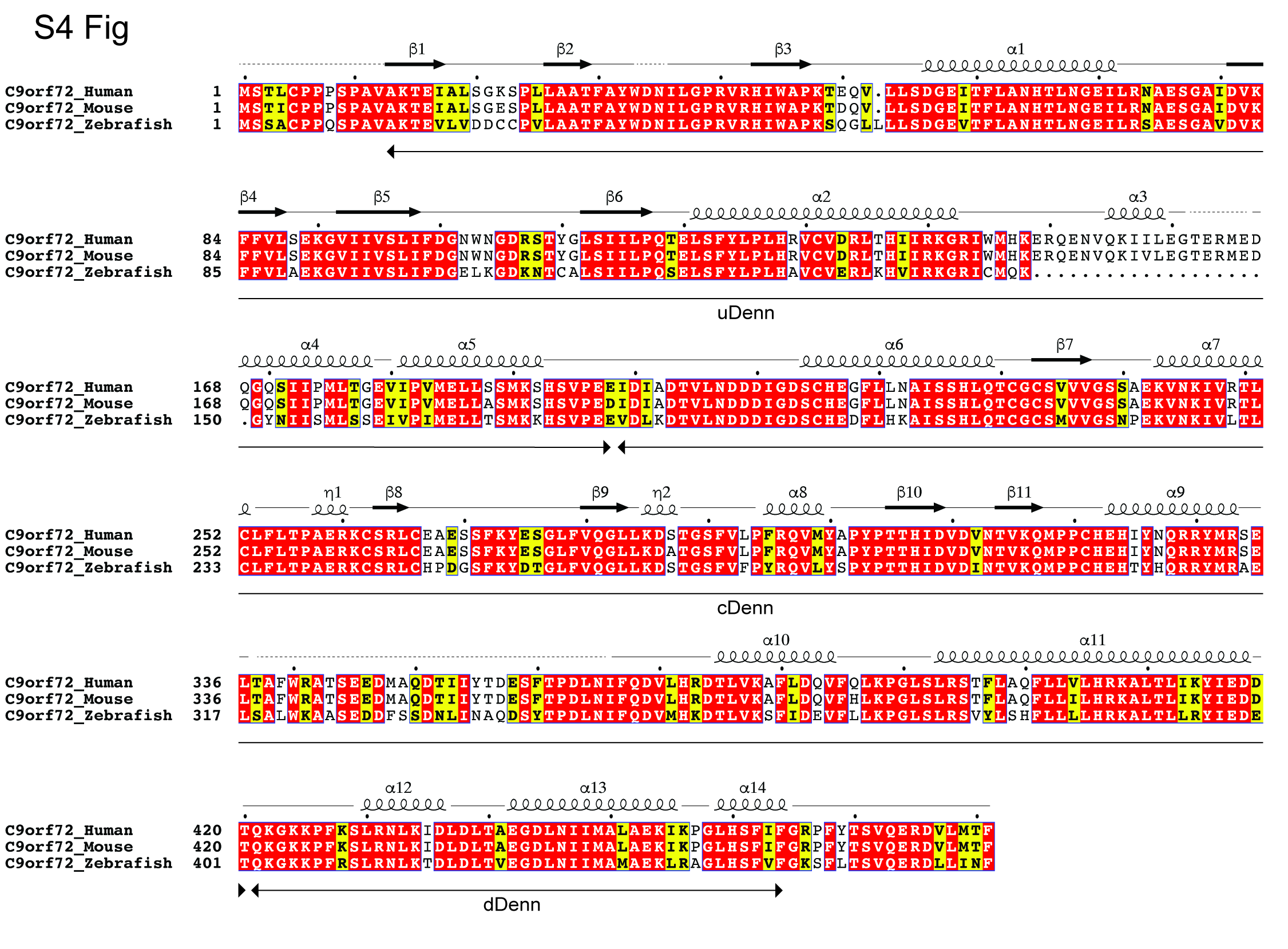

Supplement: S4 Fig — Alignment of human, mouse, and zebra danio (zebrafish) C9orf72 protein sequences. Residues that are observed in the EM map are indicated on top of each row either by the corresponding secondary structure or by a solid line. The residues that are not visible in the EM map are marked by dotted lines. Denn domains are indicated below the sequences. cDenn, central Denn; dDenn, downstream Denn; Denn, differently expressed in normal and neoplastic cells; EM, electron microscopy; uDenn, upstream Denn. (TIF) [file pbio.3001344.s004.tif]

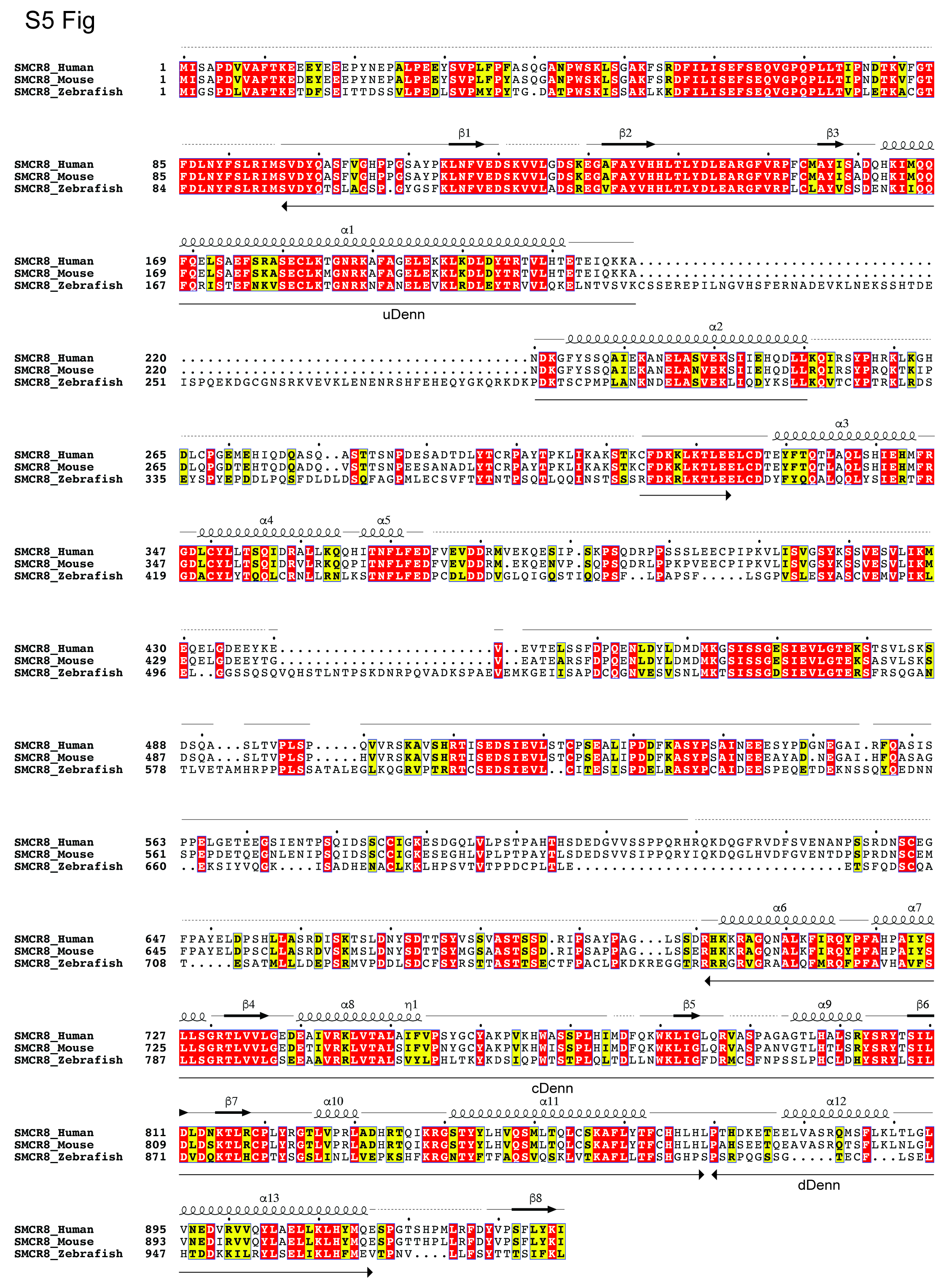

Supplement: S5 Fig — Alignment of human, mouse, and zebra danio (zebrafish) SMCR8 protein sequences. Residues that are observed in the EM map are indicated on top of each row either by the corresponding secondary structure, or by a solid line. The residues that are not visible in the EM map are marked by dotted lines. Denn domains are indicated below the sequences. cDenn, central Denn; dDenn, downstream Denn; Denn, differently expressed in normal and neoplastic cells; EM, electron microscopy; uDenn, upstream Denn. (TIF) [file pbio.3001344.s005.tif]

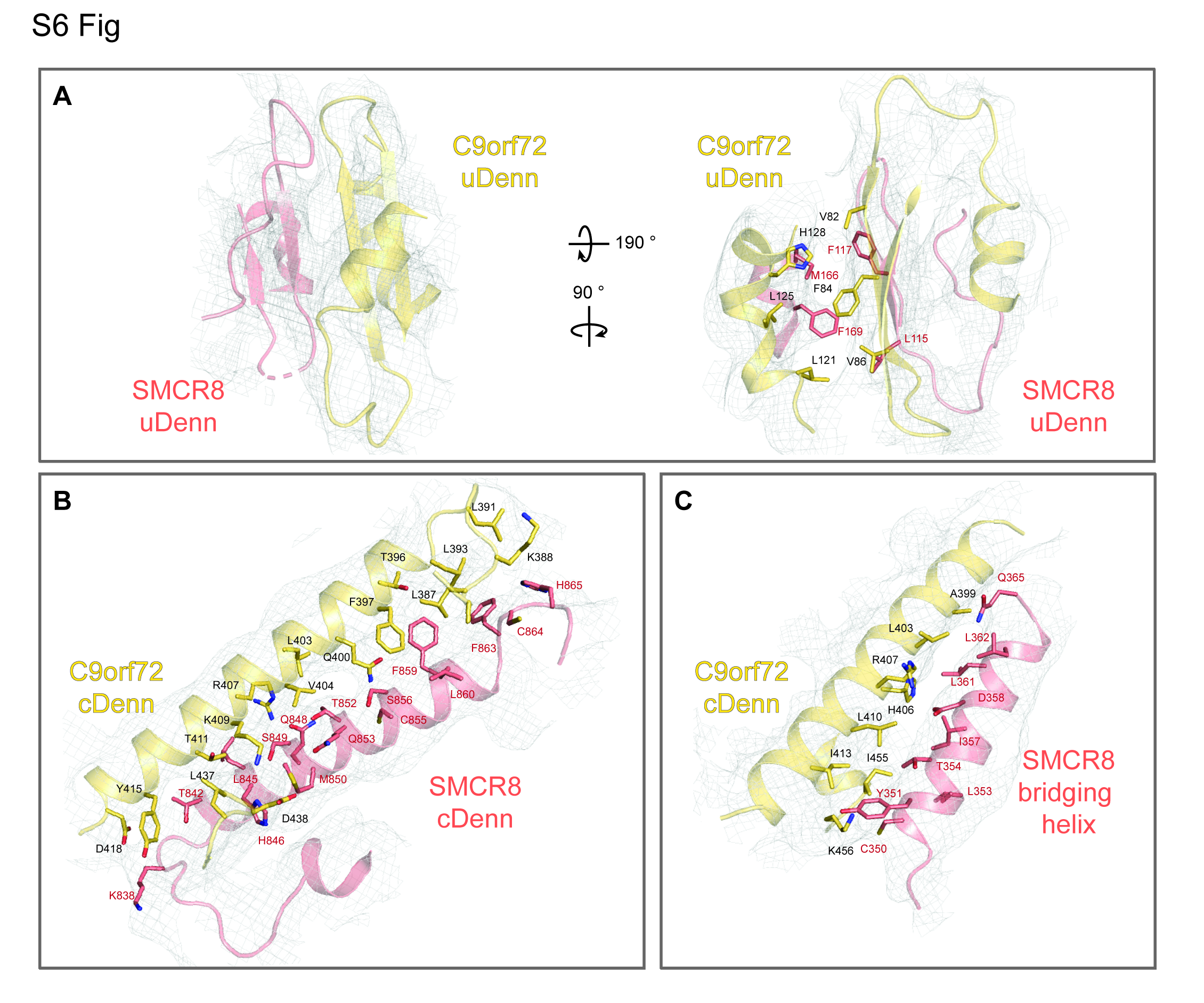

Supplement: S6 Fig — (A–C) Cryo-EM map density at the interaction interfaces of the C9orf72-SMCR8N-C (yellow orange and salmon pink) protein complex, which are shown in Fig 2B–2D, respectively. The interacting residues are labeled and displayed as sticks. cDenn, central Denn; cryo-EM, cryo-electron microscopy; dDenn, downstream Denn; Denn, differently expressed in normal and neoplastic cells; uDenn, upstream Denn. (TIF) [file pbio.3001344.s006.tif]

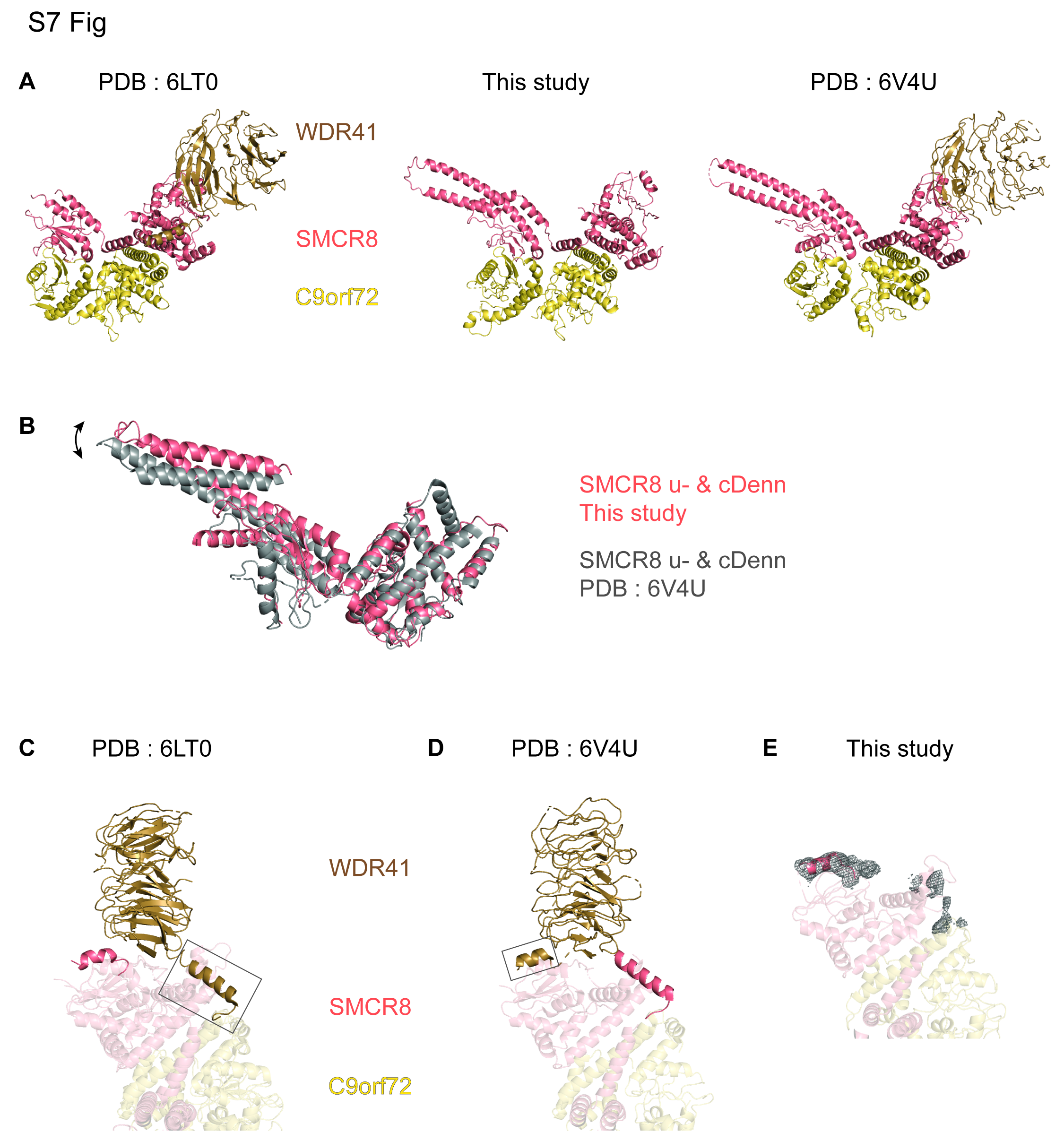

Supplement: S7 Fig — (A) Structures of the C9orf72 complex determined by Tang and colleagues (Left, PDB: 6LT0) [33], this study (Center, PDB: 7O2W) and Su and colleagues (Right, PDB: 6V4U) [34]. (B) Relative orientation of the uDenn domain of SMCR8 with respect to the cDenn domain of SMCR8 in the structure determined in this study (salmon pink) and by Su and colleagues (dark gray). Arrow indicates the change in relative orientation. (C) Position of WDR41 (brown) and the helix of SMCR8 (AA 795–806) (salmon pink) in the structure of C9orf72 complex determined by Tang and colleagues. The position of the C-terminal helix of WDR41 is boxed. (D) Position of WDR41 (brown) and the helix of SMCR8 (AA 363–383) (salmon pink) in the structure of C9orf72 complex determined by Su and colleagues. The position of the C-terminal helix of WDR41 is boxed. (E) Map densities determined in this study in the regions corresponding to the helix of SMCR8 (AA 795–806) (salmon pink) and the C-terminal helix of WDR41 (brown) modeled by Tang and colleagues are shown at same contour level. AA, amino acid; cDenn, central Denn; Denn, differently expressed in normal and neoplastic cells; PDB, Protein Data Bank; uDenn, upstream Denn. (TIF) [file pbio.3001344.s007.tif]

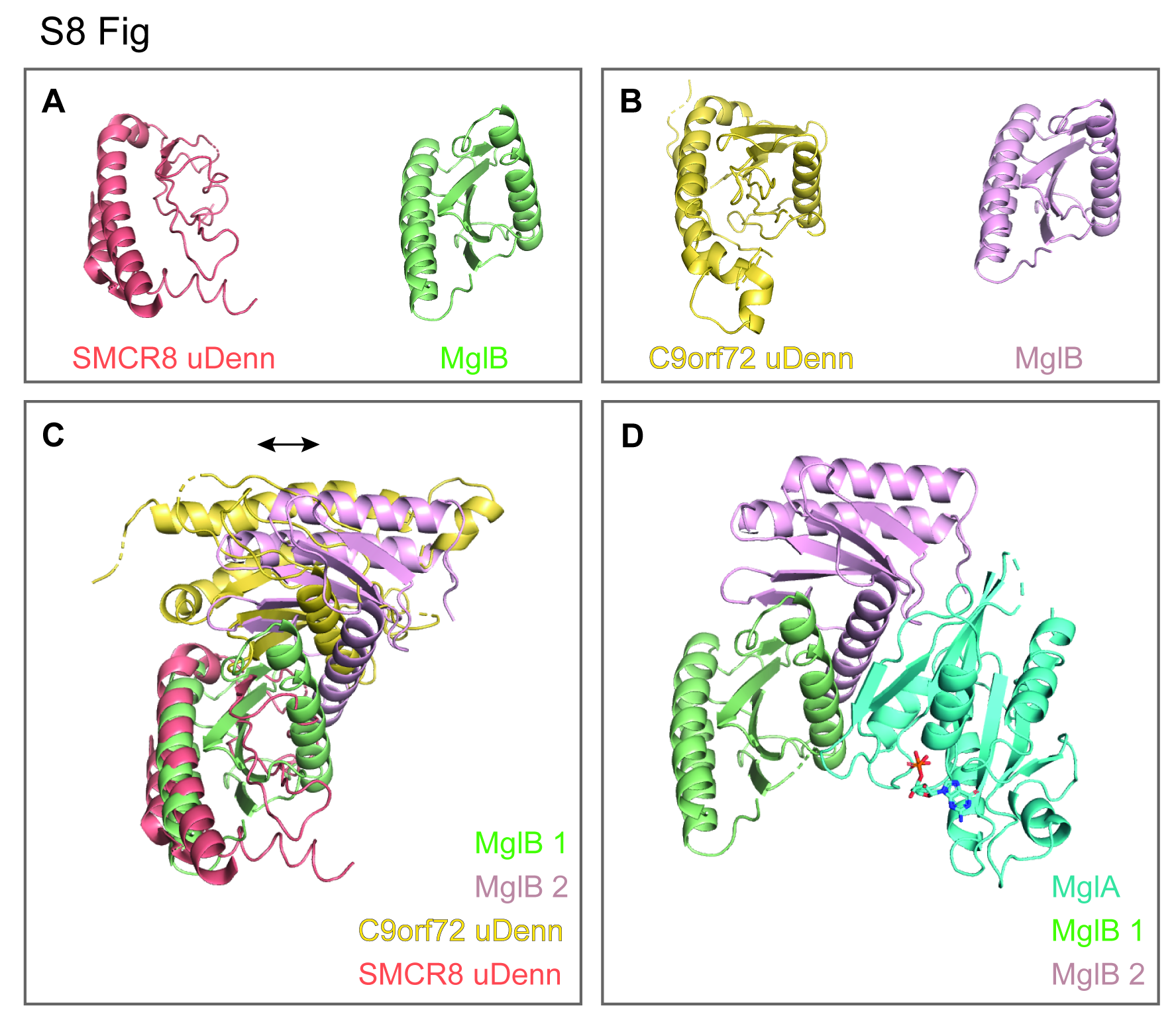

Supplement: S8 Fig — (A) uDenn domain of SMCR8 (left, salmon pink) and MglB (right, light green, PDB: 3T12) [38] are shown in similar orientation. (B) uDenn domain of C9orf72 (left, yellow orange) and MglB (right, light pink, PDB: 3T12) [38] are shown in similar orientation. (C) Superposition of MglB monomer (light green) on the uDenn domain of SMCR8 (salmon pink). The difference in orientation of C9orf72 (yellow orange) and the second MglB (light pink) monomer is indicated by an arrow. (D) Structure of MglA (cyan)-MglB (light green and light pink) complex is shown (PDB: 3T12) [38]. Denn, differently expressed in normal and neoplastic cells; PDB, Protein Data Bank; uDenn, upstream Denn. (TIF) [file pbio.3001344.s008.tif]

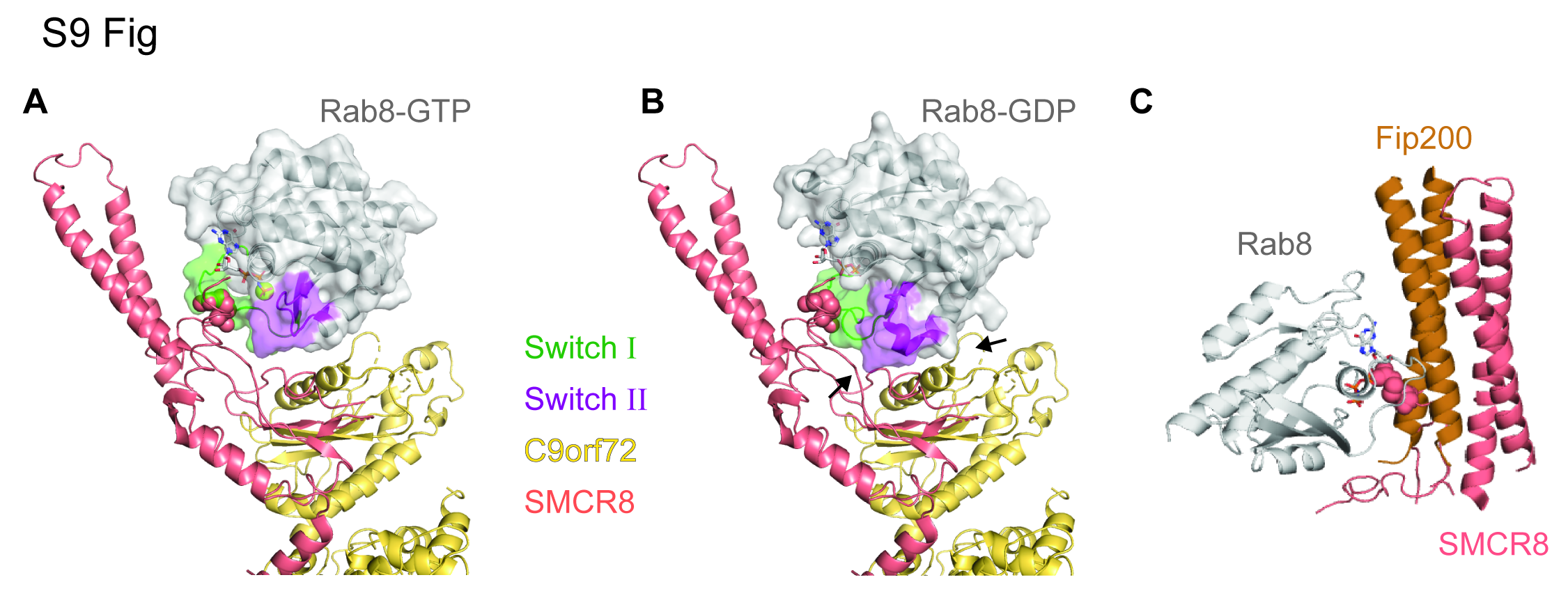

Supplement: S9 Fig — (A) Modeling of Rab8-GTP (light gray, PDB: 4LHW) [68] binding to the C9orf72-SMCR8 complex (yellow orange, salmon pink). The Switch I (green) and Switch II (magenta) of Rab8 are indicated. GTP is shown as sticks. Catalytic Arg 147 is shown in sphere representation. (B) Modeling of Rab8-GDP (light gray PDB: 4LHY) [68] binding to the C9orf72-SMCR8 complex (yellow orange, salmon pink). The Switch I (green) and Switch II (magenta) of Rab8 are indicated. GDP is shown as sticks. Clashes of the switch regions with the C9orf72-SMCR8 complex are indicated by arrows. Catalytic Arg 147 is shown in sphere representation. (C) Modeling of coiled-coil region of FIP200 (brown, PDB: 6GMA) [40] and Rab8 (light gray) with the coiled-coil region of SMCR8 (salmon pink). Catalytic Arg 147 is shown in sphere representation. PDB, Protein Data Bank. (TIF) [file pbio.3001344.s009.tif]

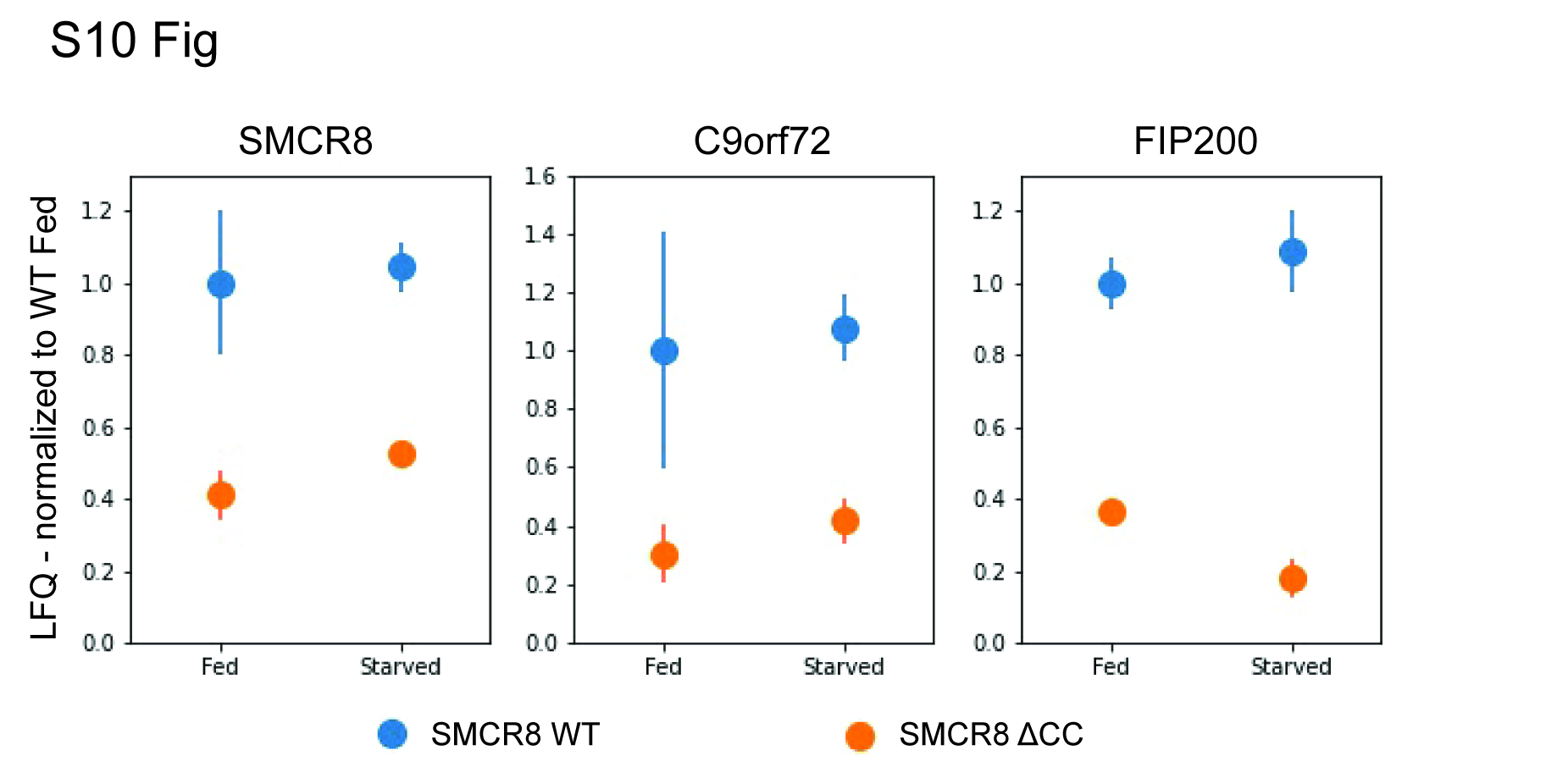

Supplement: S10 Fig — Mean fold changes in the LFQ values of SMCR8 (left), C9orf72 (center), and FIP200 (right) (normalized to the WT Fed condition) are shown for both SMCR8 Fl (WT, blue) and SMCR8ΔCC (ΔCC, orange) pull-down experiments. Standard error of the mean is shown. Student t test: C9orf72 WT Fed/Starved, p = 0.893; C9orf72 ΔCC Fed/Starved, p = 0.506; SMCR8 WT Fed/Starved, p = 0.875; SMCR8 ΔCC Fed/Starved, p = 0.265; FIP200 WT Fed/Starved, p = 0.619; FIP200 ΔCC Fed/Starved, p = 0.056; N = 3. The LFQ intensities and the normalized LFQ values can be found in S2 Data. CC, coiled coil; Fl, full-length; LFQ, label-free quantification; WT, wild-type. (TIF) [file pbio.3001344.s010.tif]
