# Supplementary material for: Structure of the human C9orf72-SMCR8 complex reveals a multivalent protein interaction architecture
Source: PLoS Biol. 2021 Jul 23;19(7):e3001344. doi: 10.1371/journal.pbio.3001344 (PMC8336837; doi:10.1371/journal.pbio.3001344)
Supplement: S1 Table — (PDF) [file pbio.3001344.s011.pdf]

S1 Table: Data collection and model validation statistics

**Data collection**	
Dataset 1	1708
Dataset 2	4097
Dataset 3	2489 (pentylamine)
Total Dose ($e^-/Å^2$)	50
Voltage	300
Pixel size	0.86
Defocus range (μm)	-0.3 to -1
Volta phase plate	+
**Refinement**	
No. of Particles	284568
B-factor	75
Resolution (Å)	3.8
No. Protein atoms	6436
No. Protein residues	841
r.m.s. deviations	
Bond lengths (Å)	0.006
Bond angles (°)	0.962
Clash Score	27.44
MolProbity Score	2.6
Mean B-factor	228.51
Map to Model (CC mask)	0.72
dFSC model (masked) (0.143/0.5) (Å)	3.4/4.2

dFSC model (unmasked) (0.143/0.5) (Å)	3.4/4.2
**Ramachandran**	
Favored	84.11
Allowed	15.89
Outliers	0
