# Supplementary material for: Structure of the human C9orf72-SMCR8 complex reveals a multivalent protein interaction architecture
Source: PLoS Biol. 2021 Jul 23;19(7):e3001344. doi: 10.1371/journal.pbio.3001344 (PMC8336837; doi:10.1371/journal.pbio.3001344)

Gel 1

Marker and peak fraction F3 are shown in the lower panel of Fig 1A.
Non-included lanes are marked with an 'X'.
Full gel is shown in S1A Fig.

Uncropped Coomassie stained SDS-PAGE gel of fractions (F1 - F7) collected during size-exclusion chromatography of affinity purified Fl-C9orf72 - Fl-SMCR8 - Fl-WDR41 complex.

Abbreviations: M, marker; MW, molecular weight; F, fraction; V, void

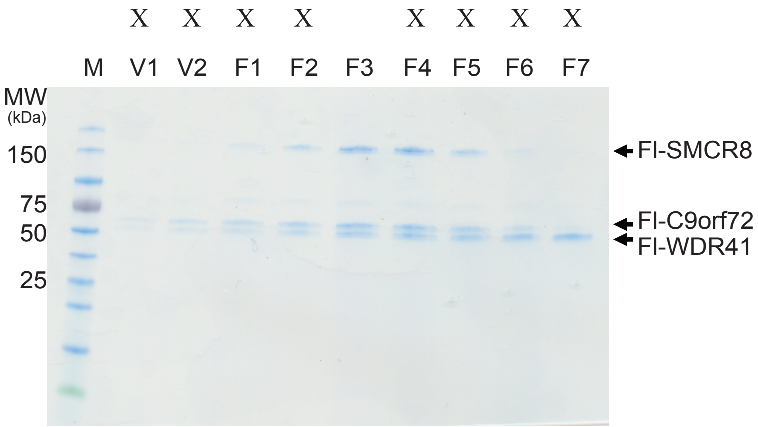

Gel 2

Marker and peak fraction F4 are shown in the lower panel of Fig 1B.
Non-included lanes are marked with an 'X'.
Full gel is shown in S1C Fig.

Uncropped Coomassie stained SDS-PAGE gel of fractions (F1 - F6) collected during size-exclusion chromatography of affinity purified Fl-C9orf72 - SMCR8$_{N-C}$ complex.

Abbreviations: M, marker; MW, molecular weight; F, fraction

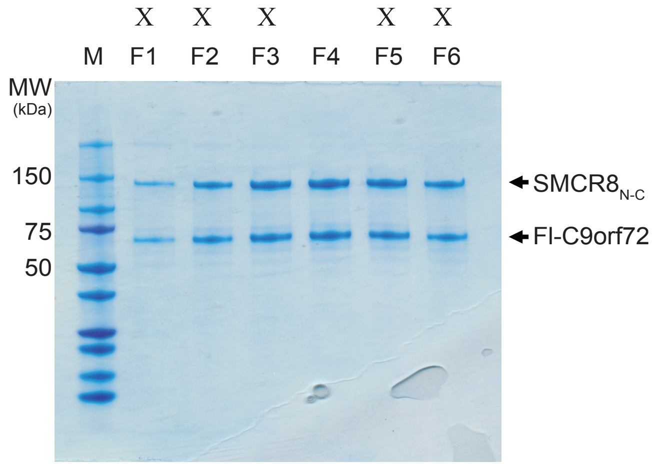

Gel 3

S1B Fig was generated from this gel.

Uncropped Coomassie stained SDS-PAGE gel analyzing the peak fraction of the proteolyzed full length trimeric C9orf72 complex obtained after size-exclusion chromatography.
The C9orf72 complex was eluted in one peak fraction, which was then analyzed on SDS-PAGE gel, using various protein concentrations. Bands, labelled from 1-10, were subsequently excised and analyzed by mass spectrometry. The identity of each band is:
1. Fl-SMCR8, 2. Fl-C9orf72, 3. Fl-WDR41, 4. Hsp70, 5. Fl-C9orf72 and $SMCR8_N$, 6. parts of SMCR8 and Fl-WDR41, 7. $SMCR8_N$ and $SMCR8_C$, 8. $SMCR8_C$, 9. central SMCR8, 10. peptides from all the proteins.

Abbreviations: M, marker; MW, molecular weight

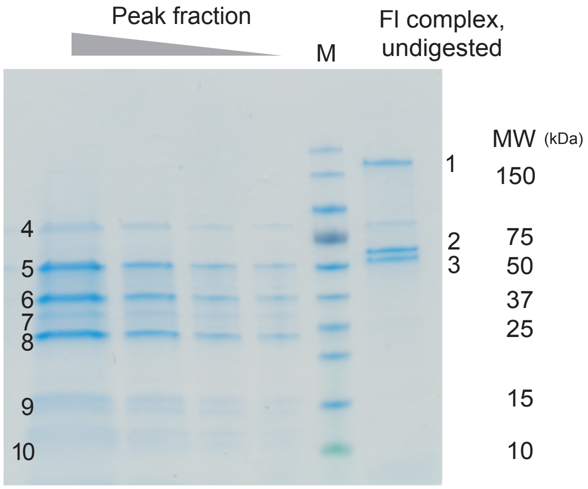

Gel 4

Fig 3A top panel was generated partly from this gel.
Non-included lanes are marked with an 'X' .

Uncropped Coomassie stained SDS-PAGE gel analyzing the input of the pulldown assay of purified WDR41 with the binary complex containing GST-C9orf72 and Fl-SMCR8 or $SMCR8_N$.
GST alone was used to assess background binding of WDR41 to the tag.
Two different GST-pulldowns (1 and 2) were carried out, which differ in WDR41 that came from two different purifications. For Fig 3A, pulldown 2 was used, which in this gel corresponds to input 2.
Used lanes are numbered equally as in Fig 3A top (left part).

Abbreviations: M, marker; MW, molecular weight

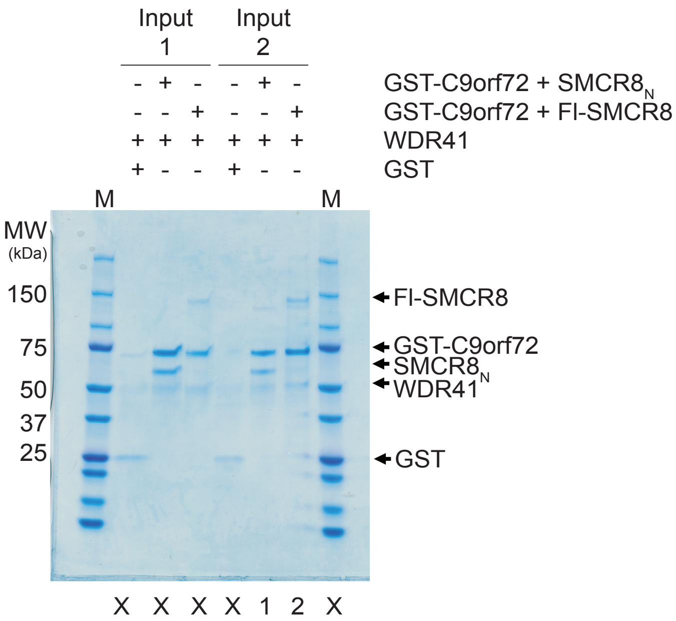

Gel 5

Fig 3A top panel was generated partly from this gel.
Non-included lanes are marked with an 'X' .

Uncropped Coomassie stained SDS-PAGE gel analyzing the pulldown assay of purified WDR41 with the binary complex containing GST-C9orf72 and Fl-SMCR8 or $SMCR8_N$.
GST alone was used to assess background binding of WDR41 to the tag.
Two different GST-pulldowns (1 and 2) were carried out, which differ in WDR41 that came from two different purifications. For Fig 3A, pulldown 2 was used.
Used lanes are numbered equally as in Fig 3A top (right part).

Abbreviations: M, marker; MW, molecular weight

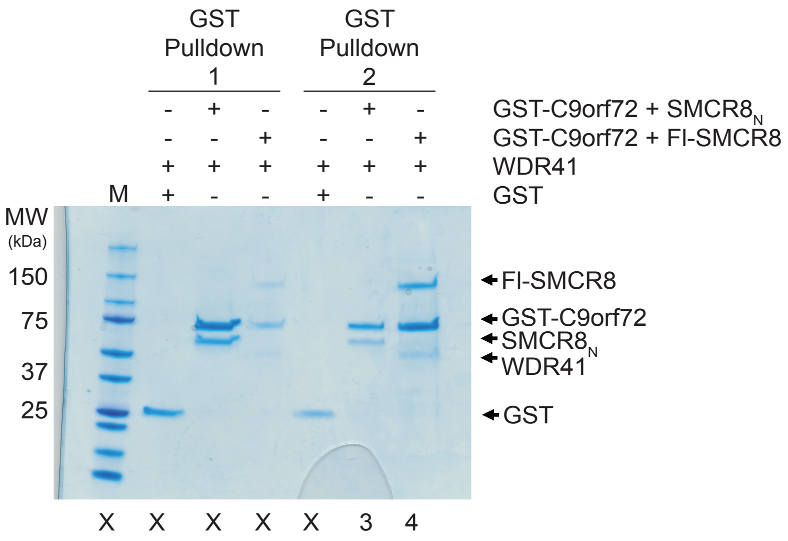

Gel 6

Fig 3A bottom panel was generated from this gel.
Non-included lanes are marked with an ‘X’ .

Uncropped Coomassie stained SDS-PAGE gel analyzing the pulldown assay of purified WDR41 with the binary complex containing SUMO-C9orf72 and C-terminal MBP-tagged SMCR8$_{N-C}$.
MBP alone was used to assess background binding of WDR41 to the tag (corresponds to lane 2 and 4).

Used lanes are numbered equally as in Fig 3A bottom.

Abbreviations: M, marker; MW, molecular weight

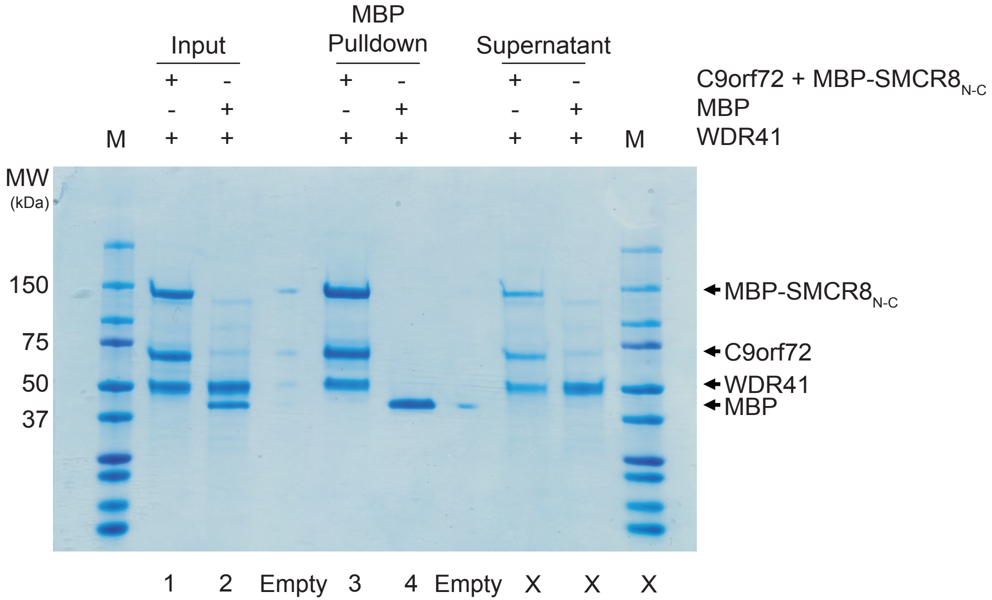

Supplement: S1 Raw Images — (PDF) [file pbio.3001344.s014.pdf]
