# Supplementary material for: Structure of the human C9orf72-SMCR8 complex reveals a multivalent protein interaction architecture
Source: PLoS Biol. 2021 Jul 23;19(7):e3001344. doi: 10.1371/journal.pbio.3001344 (PMC8336837; doi:10.1371/journal.pbio.3001344)
Supplement: Attachment — Submitted filename: Comments.docx [file pbio.3001344.s015.docx]

The authors determined the human C9ORF72-SMCR8 complex by cryo-EM single particle method and identified the coiled-coil region of SMCR8 which acts as an interaction platform for FIP200. There are several problems which should be further clarified before its publication.

**Major point:**

a, The C9ORF72-SMCR8-WDR41 trimeric complex has been solved at 3.2 Å and 3.8 Å by Qi’s and Hurley’s group, the authors failed mention those important progress. Does the C9ORF72-SMCR8 dimer has any structural difference with C9ORF72-SMCR8-WDR41 complex?

b, Description for the process of model building is not clear. 3.8 Å resolution of cryo-EM map is difficult for de-novo model building, were there any coordinates served as the initial model? Density map figures for residues in the best region should be provided to help the audiences ensure the residues assignment.

c, Description for the cryo-EM data processing is not clear, and the cryo-EM map seems problematic. The cryo-EM map in the figure seems weird, not reach 3.8 Å and suffers from overfitting. Local resolution map in Figure S2F shows most region reach 3 Å, but actually is not. Does any improvement after focus refinement at small region in the Figure S2?

**Minor points:**

a, model validation curves should be provided for all complexed.

b, Contents of Table S1 has serval errors. For example, Final particle numbers and B-factors (Lower resolution usually has a larger B-factor, 4.4 Å resolution is around -200 ). Clash score is higher, the model should be further improved. Molprobility Score should be provided.
