# Supplementary material for: Structure of the human C9orf72-SMCR8 complex reveals a multivalent protein interaction architecture
Source: PLoS Biol. 2021 Jul 23;19(7):e3001344. doi: 10.1371/journal.pbio.3001344 (PMC8336837; doi:10.1371/journal.pbio.3001344)
Supplement: Attachment — Submitted filename: Response to Reviewers.docx [file pbio.3001344.s016.docx]

## Point by point response to the reviewers

We thank all of the reviewers for the time they invested to improve our manuscript and for their helpful comments and suggestions.

## Reviewer 1 comments

In this work, Norpel and collaborators, solved the structure of the C9ORF72:SMCR8 complex by Cryo-EM. Overall, this work is well performed, clear, well written and use state if the art approaches and techniques. However, identical cryo-EM structures (PDB 6LTO and 6V4U) have been recently reported by two other groups (Tang et al., PNAS May 2020; Su et al., September 2020). Furthermore, Tang and Su studies provide additional data such as the structure of WDR41 associated to the C9:S8 complex, biochemical evidences of a GTPase activating activity for SMCR8, etc; data which are absent from the present work.
Judging the relevance for publication of a well-performed work, but that just get scooped, is always a difficult task, both for Referees and for Editors. To my personal opinion, and trying to be as fair as possible, while the cryo-EM structure of the authors is nicely done and relevant to the C9 field, there is too little novel data and information to accept this work for publication (at least in the present state).

At minima, cryo-EM data should be align to 6LTO and 6V4U structures to discuss similarities and differences. Why a large part of SMCR8 (aas 400 to 700 ?) is "invisible" to cryo-EM in all three studies should be discussed.

- We thank the reviewer for suggesting additional analysis between all the C9orf72-SMCR8 structures and have now added a comparison with the other published structures (Fig S7) and discussed them further in the result section. Importantly, our structure helps to resolve a discrepancy in the model building of WDR41 that was interpreted to belong to different regions of SMCR8 in the other structures.
- Tang *et al.* and our study have not included most of this central region (aa 400 to 700) in the SMCR8 construct that was used for expression. In our hands, this mostly disordered region affected the homogeneity of the complex and led to strong aggregation upon purification. While Su *et al*. used full length SMCR8 in their structural studies, however, they also do not see this region probably due to the fact that it is flexible as this region does not contribute to the core structure of Denn domains but connects the u- and the c-Denn domains. Consequently, it is difficult to hypothesize what its function may be at this time.

To bring some novelties, if SMCR8 is a GAP protein for ARF1 (Su et al) or RAB8 (Tang et al), maybe the authors could try to model these interaction and dock ARF1 or RAB8 proteins (for which structure are available) in their C9:S8 model.

- We thank the reviewer for this suggestion and have now modelled RAB8 GTPases binding to C9orf72-SMCR8 complex and discussed them in Fig S8 and S9 based on the prokaryotic MglA+MglB complex. Modelling of the Arf GTPases was not possible due to prominent conformational change between the GDP and GTP bound states. Nonetheless, we believe the model of Rab8 with the C9orf72-SMCR8 complex will be useful for the community.

Furthermore, the proposed hypothesis of an interaction between SMCR8 usent alpha helixes with the coil coil domain of FIP200 (RB1CC1) should not be stated without being experimentally tested.

- We have experimentally tested the binding of FIP200 to SMCR8 wildtype and SMCR8 Δcoiled-coil using pull-down mass spectrometry and label free quantification and have now included this data in Fig 5C, D, Fig S10 and Table S2. Using SMCR8 Δcoiled-coil, we found a decrease in the interaction with FIP200 compared to SMCR8 WT in starved cells. Interestingly, we also find that WDR41 binds more strongly to SMCR8 Δcoiled-coil, which highlights an interesting interplay between different regions of SMCR8 and their binding partners. How the interaction between these different factors is coordinated and how this enables the C9orf72-SMCR8 complex to perform its multiple functions will be a key question for future research.

## Reviewer 2 comments

The authors determined the human C9ORF72-SMCR8 complex by cryo-EM single particle method and identified the coiled-coil region of SMCR8 which acts as an interaction platform for FIP200. There are several problems which should be further clarified before its publication.

**Major point:**

a, The C9ORF72-SMCR8-WDR41 trimeric complex has been solved at 3.2 Å and 3.8 Å by Qi’s and Hurley’s group, the authors failed mention those important progress. Does the C9ORF72-SMCR8 dimer has any structural difference with C9ORF72-SMCR8-WDR41 complex?

- We find this comment surprising since we discussed the structure from Qi’s and Hurley’s group and included their major findings in our previous manuscript and it was never our intent to not mention their important work, so we thank the reviewer for pointing out that this was not clear previously. To further highlight the similarities and differences of their structures with the one described in this study, we have now included an additional figure (Fig S7) and added additional discussion of the three structures in the text. Importantly, our structure helps to resolve a discrepancy in the model building of WDR41 that was interpreted to belong to different regions of SMCR8 in the other structures.

b, Description for the process of model building is not clear. 3.8 Å resolution of cryo-EM map is difficult for de-novo model building, were there any coordinates served as the initial model? Density map figures for residues in the best region should be provided to help the audiences ensure the residues assignment.

- Despite the moderate resolution of our EM data, it was indeed possible to *de novo* build the structure of C9orf72-SMCR8 and we did not use the published structures for model-building. In our map, most of the helices are well-resolved and have side chain density to initiate model-building and sequence assignment as shown in Fig S3C. Furthermore, we were able to take advantage of other DENN domain containing proteins (DENN1b and Lst4) to help guide model building. Density map figures have now been included in Fig S3A and S3C.

c, Description for the cryo-EM data processing is not clear, and the cryo-EM map seems problematic. The cryo-EM map in the figure seems weird, not reach 3.8 Å and suffers from overfitting. Local resolution map in Figure S2F shows most region reach 3 Å, but actually is not. Does any improvement after focus refinement at small region in the Figure S2?

- Our data analysis suggested that the protein complex in this study adopts preferential orientations under cryo conditions. We analyzed the orientation distribution of the data according to the method described by Naydenova and Russo (Measuring the effects of particle orientation to improve the efficiency of electron cryomicroscopy, *Nature Communications* 8 (2017)) and quantified the amount of anisotropy in the 3D reconstructions by the cryoEF coefficient (a value higher than 0.7 is considered isotropic).
- Thus, we have reprocessed our dataset to generate an additional map (4.37Å) with the intent to minimize the impact of preferential orientation in our 3D reconstructions. To do so, we systematically removed particles that were contributing to the over-represented views. We achieved this in two ways during the 2D class selection task in Relion, we limited to 20k the maximum number of particles per 2D class. We now report these coefficients below the key reconstructions in Fig S2.
- Due to the resolution anisotropy, the estimated resolutions are slightly overestimated. Nevertheless, the resolution of the map allowed to build the structure. Importantly, the quality of our map allows us to resolve the discrepancy in the position of the C-terminus of WDR41 that was modeled differently in the two other publications.

**Minor points:**

a, model validation curves should be provided for all complexed.

- We have now included a model validation curve (Fig S3B).

b, Contents of Table S1 has serval errors. For example, Final particle numbers and B-factors (Lower resolution usually has a larger B-factor, 4.4 Å resolution is around -200). Clash score is higher, the model should be further improved. Molprobility Score should be provided.

- We have further refined the model and included its statistics in Table 1.
