# Supplementary material for: Structure of the human C9orf72-SMCR8 complex reveals a multivalent protein interaction architecture
Source: PLoS Biol. 2021 Jul 23;19(7):e3001344. doi: 10.1371/journal.pbio.3001344 (PMC8336837; doi:10.1371/journal.pbio.3001344)
Supplement: Attachment — Submitted filename: Response-to-reviewers.docx [file pbio.3001344.s017.docx]

## Response to the reviewers

We thank Reviewer 1 for the additional time they invested to improve our manuscript and for their helpful comments and suggestions.

## Reviewer 1 comments

Rev. 1:
The authors adequally addressed my comments. 

As a minor point, modelisation of the C9 complex with an ARF (ARF1 ? ARF6 ?) versus RAB8 could have been presented and discussed as both RAB8 and ARF proteins were found as potential interactants / targets of this complex, etc.

We thank the reviewer for this suggestion, however, modelling of the Arf GTPases was not possible due to a prominent conformational change between the GDP and GTP bound states. Furthermore, the clash scores of both the GDP and GTP bound conformations when modelled on our C9orf72-SMCR8 complex structure were similar and, therefore, we believe this comparison would not be informative. Additionally, the Hurley lab has recently published a preprint, which appeared after our revision was submitted, of ARF1+GDP bound to C9orf72-SMCR8-WDR41 so this structure will be available to the community soon.

(https://www.biorxiv.org/content/10.1101/2021.04.13.439632v1).
